# miRNA in Molecular Diagnostics

**DOI:** 10.3390/bioengineering9090459

**Published:** 2022-09-09

**Authors:** Maja Matulić, Paula Gršković, Andreja Petrović, Valerija Begić, Suzana Harabajsa, Petra Korać

**Affiliations:** 1 Division of Molecular Biology, Department of Biology, Faculty of Science, University of Zagreb, 10000 Zagreb, Croatia; 2 Institute of Clinical Pathology and Cytology, Merkur University Hospital, 10000 Zagreb, Croatia; 3 Primary School “Sesvetski Kraljevec”, 10361 Sesvetski Kraljevec, Croatia; 4 Department of Pathology and Cytology, Division of Pulmonary Cytology Jordanovac, University Hospital Centre Zagreb, 10000 Zagreb, Croatia

**Keywords:** miRNA, malignant tumors, viral infections, diagnostics

## Abstract

MicroRNAs are a class of small non-coding RNA molecules that regulate gene expression on post-transcriptional level. Their biogenesis consists of a complex series of sequential processes, and they regulate expression of many genes involved in all cellular processes. Their function is essential for maintaining the homeostasis of a single cell; therefore, their aberrant expression contributes to development and progression of many diseases, especially malignant tumors and viral infections. Moreover, they can be associated with certain states of a specific disease, obtained in the least invasive manner for patients and analyzed with basic molecular methods used in clinical laboratories. Because of this, they have a promising potential to become very useful biomarkers and potential tools in personalized medicine approaches. In this review, miRNAs biogenesis, significance in cancer and infectious diseases, and current available test and methods for their detection are summarized.

## 1. MicroRNA Biogenesis

MicroRNAs (miRNAs) are a class of small RNAs, with an average length of 22 nucleotides (nts). They are highly conserved non-coding RNA molecules that regulate gene expression in many organisms, as well as in the human genome. By patrolling the genome and transcriptome, miRNAs play important roles in many cellular and developmental processes of eukaryotic organisms [1]. The first discovered miRNA, lin-4, controls developmental timing in the nematode *Caenorhabditis elegans* by blocking gene *lin-14*, which encodes proteins that are crucial for the continuation of developments after the larvae stage [2,3]. A few years later, another gene, let-7, which encodes 21 nts-long molecule RNA important in the development of the same species, was discovered [4]. The number of known miRNAs has since expanded substantially [5,6].

Considering that small changes in miRNA levels can disturb the regulation of different target genes, the adequate control of miRNA biogenesis is essential for the maintenance of cell homeostasis. miRNA biogenesis is subjected to complex regulation at both transcriptional and post-transcriptional levels in order to yield functionally mature miRNAs [7]. Most miRNA genes are located in protein non-coding regions of the genome or within introns of genes that code proteins. miRNA expression is regulated independently of the expression of a host gene through their own promoters [8]. RNA polymerase II and, in some cases, RNA polymerase III transcribe miRNA genes. miRNA genes are in most cases transcribed into miRNA primary transcripts (pri-miRNAs), which form short hairpins because of imperfect complementarity and are more than 1000 nts long. Pri-miRNAs, transcribed by RNA polymerase II, contain 7-methylguanylate cap at the 5′-end and poly-A tail at the 3′-end [9,10].

**Figure 1:**
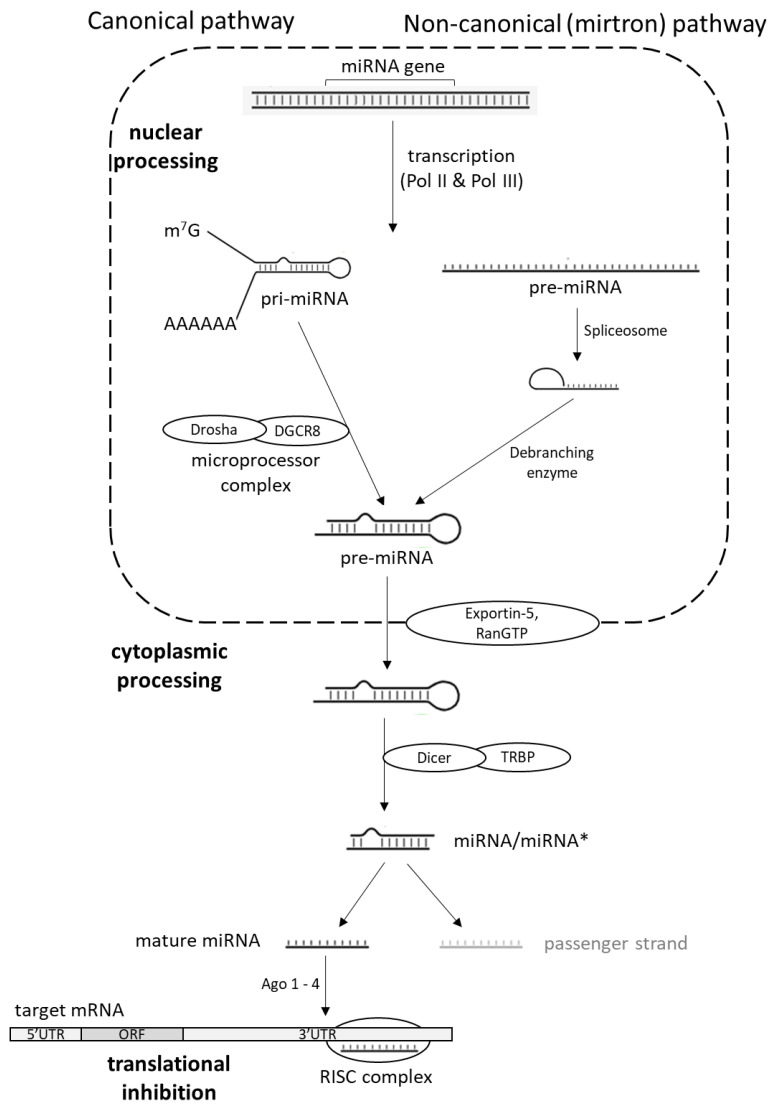
miRNA biogenesis pathways.

The biogenesis of miRNA represents a series of sequential processes forming canonical or non-canonical pathways (Figure 1). However, most miRNAs are known to be synthesized by a canonical pathway. In canonical pathways, miRNAs pass through two sequential maturation steps. The first step involves enzyme Drosha (RNase III enzyme) in the nucleus and a second enzyme, Dicer, in the cytoplasm. In the nucleus, pri-miRNAs are processed into pre-miRNAs. The RNA stem-loop is recognized by the nuclear protein DGCR8 (Di-George Syndrome Critical Region 8) that binds to Drosha, forming a microprocessor complex that cleaves pri-miRNA in order to form pre-miRNA. Pre-miRNAs are ~65 nts in length and are observed to be in formation with two-nucleotide-long 3′ overhang [11,12]. A transmembrane protein, exportin 5, recognizes 3′ overhang ends on pre-miRNAs and, with cofactor RanGTP, transfers them to the cytoplasm. After they are exported from the nucleus by the exportin 5/RanGTP complex, pre-miRNAs in cytoplasm are processed by Dicer. Enzyme Dicer contains two RNAse III catalytic domains, a double-strand RNA-binding domain and the PAZ domain, that bind the 3ʹ-end of small RNAs and RNA helicase/ATPase domain [12,13,14,15]. Through its helicase domain, Dicer associates with cofactor TRBP (TAR-RNA binding protein). The Dicer-TRBP complex removes the terminal loop of the pre-miRNA to release a mature double -strand miRNA (dsRNA) [16], although TRBP does not seem to be essential for Dicer-mediated pre-miRNA processing [17]. Generated dsRNAs, approximately 22 nts long, are named miRNA/miRNA* (miRNA-guide strand, miRNA*-passenger strand) or miR-3p/miR-5p, referring to the direction of the functional miRNA. The 3p strand arises from the 3′-end of the pre-miRNA hairpin, while the 5p strand originates from the 5′-end. One of the strands of dsRNA is the mature miRNA guide, and the other is the complementary passenger strand. The origin of mature miRNA from either 5p or 3p strand depends on the cell type, cellular environment, thermodynamic stability, and many other factors. Generally, the guide strand has lower 5′ stability, and it is introduced into RISC complex (RNA-induced silencing complex), which contains the Argonaute (AGO 1-4) protein family and acts as a post-transcriptional regulator. Considering that the RISC complex plays an important role in the execution of miRNA-based silencing, miRNA’s function is to guide the RISC complex to complementary sequences on target mRNAs. In most cases, miRNAs interact with the complementary 3′ UTR region of target mRNAs to induce mRNA degradation and translational inhibition [18,19,20,21].

A non-canonical pathway uses different combinations of proteins in comparison with a canonical pathway. While Dicer is almost always indispensable in canonical and non-canonical pathways, the microprocessor complex is only needed for canonical pathways [22]. The first non-canonical pathway discovered was the mirtron pathway. In this alternative pathway, introns are processed by spliceosomes and debranching enzymes in the nucleus, which results in miRNA hairpins directly suitable for Dicer cleavage. Such hairpins are then exported to the cytoplasm by exportin 5 (Figure 1) [23,24,25].

Mature miRNAs have important regulatory roles that ensure timely and precise usage of transcriptome in different cells. Aberrations in their function are recognized as crucial points in the pathogenesis of different diseases, particularly in cancer development and viral infections.

## 2. miRNA in Malignant Tumors

miRNAs influence the expression of numerous proteins, including the expression of tumor suppressors and protooncogenes, thus becoming oncogenes and tumor suppressors themselves. As the same miRNA can have different targets in different tissues, its function will also be different in different types of tumors, depending on the intracellular milieu and the set of proteins for which its translation is modulated. Therefore, the same miRNA can act as a tumor suppressor and an oncogene in different tumors. Even in the same tumor, the same miRNA can be involved in regulation circles with feedback loops and potentially affect both tumor suppressors and oncogenes [26]. As 50% of miRNA genes are located in regions associated with cancers, their expression is found to be deregulated in tumors [27]. miRNAs were found to be members of signaling circuits, often involving also long non-coding RNAs (lnc RNAs) and circular RNAs (cRNAs). As oncomirs and tumor suppressors, miRNAs mostly influence processes of cell proliferation and apoptosis. Examples of most investigated miRNAs in various malignant tumors and their targets are listed in Supplementary Table S1 [28,29,30,31,32,33,34,35,36,37,38,39,40,41,42,43,44,45,46,47,48,49,50,51,52,53,54,55,56,57,58,59,60,61,62,63,64,65,66,67,68,69,70,71,72,73,74,75,76,77,78,79,80,81,82,83,84,85,86,87,88,89,90,91,92,93,94,95,96,97,98,99,100,101,102,103,104,105,106,107,108,109,110,111,112,113,114,115,116,117,118,119,120,121,122,123,124,125,126,127,128,129,130,131,132,133,134,135,136,137,138,139,140,141,142,143,144,145,146,147,148,149,150,151,152,153,154,155,156,157,158,159,160,161,162,163,164,165,166,167,168,169,170,171,172,173,174,175,176,177,178,179,180,181,182,183,184,185,186,187,188,189,190,191,192,193,194,195].

As illustrated on the example of osteosarcoma [196], miRNA can influence all hallmarks of cancer: cell cycle, proliferation control mechanisms, cell migration, metastasis and invasion, autophagy, apoptosis, senescence, and differentiation. They also affect resistance to chemotherapeutics, metabolism, and immune surveillance [74,138,160,168].

Specific changes in miRNA regulation can be found in all types of tumors, but detecting those that could be used as diagnostics markers is challenging, as they are cell- or tumor-specific. Currently, besides detecting one specific miRNA, often, a set of deregulated miRNAs is correlated with a certain type of tumor. For example, Sharma and Gupta analyzed miRNAs as potential biomarkers for the diagnosis and prognosis of different types of cancer and detected 723 dysregulated miRNAs in 16 types of tumors. Forty-three miRNAs were differentially expressed in six or more types of tumors [197].

### 2.1. miRNAs in Leukemia and Lymphoma

First miRNAs found to be directly involved in tumor development were detected in leukemia patients. The involvement in proliferation and apoptosis and their roles in the signaling loops in B cell differentiation are probably the best known and understood in hematopoietic cells and their malignancies [26]. Calin discovered that the deletion of 13q14 in chronic lymphocytic leukemia (CLL) coincided with a loss of miRNA15a and miRNA16-1 (miR15/16), and in nearly 70% of CLL patients, these miRNAs were found to be either absent or epigenetically downregulated [28]. Their deletion influenced the expression of antiapoptotic proteins BCL2 and MCL1, leading to survival and resistance to chemotherapeutics, as well as cyclin D1 expression and cell proliferation [28,29,30]. As their target is also p53, their deletion leads to the development of a specific CLL subtype. miR15/miR16 cluster downregulation was also found to be involved in other types of leukemia [31].

Another miRNA gene cluster for which its expression correlated with development of hematopoietic disorders was miR17-miR92 cluster, containing six miRNAs, produced from a polycistronic transcript [32]. Its amplification was found in B cell lymphoma, T cell leukemia, and some solid tumors [32,198]. Its locus is regulated by c-Myc, and its main targets are BIM, PTEN, p21, and p57 [199]. Its deregulation compromises apoptosis and increases proliferation, cell survival, resistance to chemotherapy, and BCR signaling [34,35,36].

Numerous miRNAs were further found to be involved in normal development and leukemogenesis in both B and T lymphocytes. One of them is miR34a, which acts as a tumor suppressor and is regulated by p53. Its main targets are *FOXP1*, *ZAP-70,* genes involved in apoptosis, such as *BCL2*, and genes regulating cell cycle progression and proliferation, such as *BCL6*, *B MYB*, *CDK6*, and *AXL* [37].

While *FOXP1* is regulated by miR34a in pro/pre-B cells, miR150 regulates this gene in mature B cells. In pro-B cells, miR150 regulates Myb [26,38]. Its deletion increases BCR signaling and survival pathways involving PIK3AP1 and AKT2 and influences telomerase expression. By targeting *CXCR4* it regulates mobilization and migration of mononuclear cells [39]. Its expression is downregulated in one CLL subtype and in different types of lymphomas.

miR155 can act as both an oncogene and a tumor suppressor in different steps of B cell development, and it is another example of different types of negative feedback loops present in the signaling regulations of miRNAs [26]. It is regulated by BCR activation, its downstream targets are transcription factor Pu.1 and AID, which are involved in immunoglobulin somatic hypermutation [40], and it regulates Akt signaling, proliferation, motility, and the modulation of TGFβ pathways [41]. miR181b also targets AID, in addition to Bcl2, MCL1 and TCL1, and Akt kinases coactivator, thus influencing apoptosis, cell survival, and differentiation. This miRNA is downregulated in CLL, and its levels can correlate with disease progression in the samples of the same patient [42,43].

### 2.2. miRNA in Brain Tumors

miRNA in glioblastoma and other brain tumors were analyzed by several research groups, detecting characteristic sets of 5–10 up- and downregulated miRNAs as specific signatures that correlated with patients’ survival. These miRNAs influence MAPK, PI3K/Akt, mTOR, and Wnt signaling pathways and deregulate apoptosis and the control of proliferation [47,56]. Among miRNAs, the most analyzed miRNA in these tumor types is miR7, and it is involved in neural cell differentiation and acts as a tumor suppressor. It mainly influences targets in Akt and MAP kinase pathways, and its downregulation increases proliferation, survival, and inhibits apoptosis [48,200]. Other miRNAs often involved in glioblastoma development are miR21, miR221, and miR181 [49,50,56]. miR21 overexpression leads to the inhibition of apoptosis and increases in cell proliferation [49,50,201]. Other mentioned miRNAs are involved in PI3K/Akt regulation, Notch and p53 signaling, and DNA repair [54,56,62].

### 2.3. miRNA in Lung Cancer

miR21 [64], miR148 [77], and miR205 [65] are among the most investigated miRNAs in lung cancer, but numerous other miRNAs were also found to be deregulated in this type of tumor. Several sets of miRNAs with altered expression were also identified in lung adenocarcinoma, in addition to observing differentially expressed miRNAs in different types of lung cancer [202,203,204,205]. It was found that EGFR mutation in lung cancer cells leads to changes in the expression of 17 miRNAs, including the miR17-92 cluster [79]. These miRNAs influence proliferation, survival, resistance to chemotherapy and apoptosis, and migration and cancer cell stemness [73,206]. The main targets of deregulated miRNAs in lung tumors are Ras and Myc pathways, PTEN and PI3K/Akt signaling leading to cell proliferation, the p53 pathway, and others influencing resistance to chemotherapeutics and apoptosis [65,69,70,71]. Cell migration, proliferation, and resistance to chemotherapeutics are influenced by the interaction of miRNA with HIF and TGFβ pathway elements [63,76,158]. Other processes regulated by miRNAs in lungs are metabolism and glycolysis, as well as epithelial–mesenchymal transition (EMT) [71,73,76,207,208].

### 2.4. miRNA in Breast Carcinoma

Numerous miRNAs and signatures of deregulated miRNA were detected in breast cancer [209,210]. Among those, the most investigated are miR125b, miR145, miR21, miR155, and miR205 [81,82,211]. The main targets of deregulated miRNAs are molecules involved in MAP/AKT/STAT3 signaling pathways and those regulating cell proliferation, epithelial–mesenchymal transition, angiogenesis by targeting VEGFA, cell stemness, and resistance to chemotherapy [61,80,83,84,85,86,212].

### 2.5. miRNA in Bladder and Renal Carcinoma

In bladder cancer, deregulated miRNAs often include miR34a, miR21, and miR222, and several miRNAs are linked to migration and invasion [96,97,98]. Their targets are β catenin, CDK2, E cadherin, as well as integrin α5, influencing resistance to chemotherapy [94,100,101].

In renal cancer tissues, the main targets of deregulated miRNAs include proteins participating in proliferation, such as those in Akt and Wnt signaling, migration, invasion, and EMT [99,104,105,107,213].

### 2.6. miRNA in Colon, Hepatocellular and Gastric Carcinoma

In colon cancer, deregulated miRNAs, including miR200c, miR145, miR181, miR101, and miR21, mainly interfere with cell proliferation and migration, apoptosis, Wnt/β-catenin, and MAPK pathways [108,110,111,114,115]. In this entity, specific sets of miRNAs with prognostic and diagnostic potential were detected [214].

The most significant miRNAs involved in signaling circuits in hepatocellular carcinoma are those regulating the PI3K/Akt pathway, cell proliferation, apoptosis, invasion, EMT, and glucose metabolism [117,119,120,121,122]. They mainly act as tumor suppressors [126,215,216].

In gastric carcinoma, miRNAs also regulate cell proliferation and migration by targeting PTEN and EGFR, as well as MAPK pathways, and EZH2, which participates in chromatin remodeling [126,127,129]. Numerous miRNAs in this disease are related to resistance to apoptosis through the regulation of Bcl2 or other members of its family, angiogenesis, and resistance to chemotherapy [132,133,134,217].

In pancreatic cancer, miRNAs regulate EMT through TGFβ signaling, as well as processes of invasion and the inhibition of apoptosis [135,136].

### 2.7. miRNA in Cervical Carcinoma, Testicular Tumors, and Prostate Cancer

In cervical carcinoma, miRNAs promote tumor proliferation, migration, invasion, and influence apoptosis and chemoresistance. Examples are miR21, which influences Akt/mTOR pathway, proliferation, growth, and EMT [140]; miR375, which targets E-cadherin; and miR138, which targets EZH2, influencing chromatin remodeling [141,218]. It was observed that viral proteins E6 and E7 increase the expression of miR18a [137], influencing Hippo signaling, in human papilloma virus (HPV)-associated cervical carcinoma.

In prostate cancer, miRNAs influence proliferation, apoptosis, migration and invasion. The main targets are Akt and MAPK pathways and HIF and VEGF pathways [141,146,150,151].

In different types of testicular germ cell tumors, several miRNAs are differently expressed and vary from low expression in teratoma, medium expression in seminoma, and high expression in embryonal carcinoma. The main deregulated miRNAs are miR199-214, influencing tumor metabolism through epigenetic regulators, miR371-373 influencing p53 pathway, cell cycle regulation, Wnt/β-catenin signaling, and senescence; and miR223, influencing apoptosis and cell growth through FBXW7 [152,154,155,159,160]. Other miRNA targets are cell cycle regulators, members of the p53 pathway involved in apoptosis regulation, DNA damage sensitivity, cell differentiation, and lactate metabolism [154,158,219,220].

### 2.8. miRNA in Skin Tumors

An analysis of metastatic melanoma revealed 44 miRNAs acting as tumor suppressors and 23 as oncomirs [221]. Some of those miRNAs control the expression of MITF, transcription factor involved in differentiation, proliferation, and the survival of melanocytes, cell motility, and invasiveness [222]. Numerous miRNAs control MITF directly, and others control MITF by targeting signaling pathways regulating its expression, such as Wnt and MAP signaling. Furthermore, some miRNAs regulate cell survival and take part in chromatin remodulation [162]. Developments in invasive melanoma are linked to melanoma phenotype switching when a highly proliferative state is exchanged for invasive states characterized by its migration ability. In this state, MITF levels decrease. Phenotypic changes have similarities to EMT, and numerous miRNAs involved in the regulation of migration are deregulated. High MITF expressions also correlate with resistance to chemotherapy, and nearly 20 tumor suppressors and oncomirs are linked to this process, with most of them targeting MAP kinase and PI3K and EMT pathways [165,166]. miRNAs in melanoma also regulate escape from immune surveillance [168].

In cutaneous squamous cell carcinoma, miRNAs influence cell proliferation, invasion, and migration; and inhibit apoptosis and differentiation by targeting PTEN, members of MAP kinase, and cMyc pathways [170,171,173].

### 2.9. miRNA in Other Tumors

Aplastic thyroid cancer is a highly invasive thyroid tumor that is fast growing and resistant to chemotherapy. miRNAs influence cell proliferation, invasion and EMT, cell adhesion, differentiation, and cell stemness by targeting PTEN, CDKI, NFκB, TGFβ, Wnt pathway, and ZEB, which are proteins involved in autophagy, apoptosis, and chromatin modulation [179,181,182,185,187,223]. In a rare medullary thyroid carcinoma, specific sets of deregulated miRNAs were detected [182,189].

In osteosarcoma, most deregulated miRNAs are linked to cell proliferation and migration: targeting β-catenin and MAP kinases pathways [191,192,193]. There are miRNAs that act as oncomirs and tumor suppressors depending on the intracellular milieu of different osteosarcomas [194,195,196].

## 3. miRNA in Viral Diseases

Other than modulating many biological functions on a cellular level, such as cell proliferation and differentiation, miRNAs also modulate host immunity and viral infections by regulating the expression of more than 60% of human genes [224,225]. In addition to eukaryotic cellular miRNAs, viral miRNAs (v-miRNAs) and their functions in immune responses have been broadly studied in the past decade [225,226]. Similarly to cellular miRNAs, v-miRNAs interact with the 3′ untranslated region of the target mRNAs. This permits v-miRNAs to function as hosts’ gene regulators as well as viral gene regulators, helping viruses in staying hidden from the host’s immune system [225,226,227].

Cellular and viral miRNAs with roles in different viral infections are summarized in Supplementary Tables S2–S11 [224,225,226,228,229,230,231,232,233,234,235,236,237,238,239,240,241,242,243,244,245].

### 3.1. DNA Viruses

#### 3.1.1. Herpesviruses

The first v-miRNA, encoded by DNA Epstein-Barr virus (EBV), was discovered in 2004. EBV is known to encode over 40 v-miRNA, and more than 250 v-miRNA have been identified since, of which most are encoded by all three families of herpesviruses [226,228,238].

There are three types of alpha-herpesviruses: herpes virus simplex 1 (HSV-1), herpes virus simplex 2 (HSV-2), and varicella zoster virus (VZV) [224,226,238]. Many studies attempted to detect VZV v-miRNA as well as cellular miRNA during VZV infection, but these v-miRNA have not been identified yet [226]. HSV-1 and HSV-2 cause productive infection in the oral and genital mucosal epithelium and latent infection in the sensory neurons [224,246]. Studies conducted about those viruses are focused on detecting miRNAs that are expressed during the latent phase of viral infection [226,246]. It was discovered that not only v-miRNAs but also specific host cellular miRNAs are expressed during latent phase [224]. MiR101 and miR138 are two main cellular miRNAs that inhibit the active phase of the infection and promote HSV-1 viral latency [224]. To stay latent, HSV-1 must avoid the host’s immune system, and for that, it uses cellular miRNAs such as miR23 [224,228,238]. The infection of monocytes with HSV-1 upregulates miR132, which has a negative effect on the expression of interferon-stimulated genes. The transcriptional co-activator p300, which is essential for the initiation of antiviral immunity, is a target of miR132 [224,229].

The most known beta-herpesvirus is cytomegalovirus (HCMV). The most important cellular miRNAs that help HCMV stay in the latent phase of the infection are the miR200 family [230]. MiR200 family members bind to 3′UTR of *ULI122* mRNA and prevent its translation [224,230]. Another important cellular miRNA for which its overexpression leads to innate immune-response evasion is miR132, which has the same effect during HCMV infections as during HSV-1 infections [224,229].

EBV and Kaposi’s sarcoma-associated herpesviruses (KSHVs) are the two most studied viruses in the beta-herpesvirus family [224,226,233]. Infection with EBV induces many changes in cellular miRNAs, such as in the miR17-92 cluster, which is essential for differentiation of immune cells (B cells, T cells, NK cells, and macrophages) that are infected by the virus [224,232,233].

KSHV, also known as human-herpesvirus 8 (HHV8), can encode viral IL-6 (vIL-6) that mimics all activities of human IL-6 (hIL-6) [224,226,234,246]. Cellular miRNAs that are involved in the regulation of vIL-6 and hIL-6 through binding sites in their open reading frames (ORF) are miR608 and miR1293 [224,234]. Moreover, miR31 is upregulated in KSHV-infected lymphatic endothelial cells (LECs) [224,235].

Moreover, since the discovery of the first v-miRNA, there have been over 500 v-miRNAs that can function as gene regulators for both host and viral genes in order to regulate latency and help viruses evade hosts’ immune responses [224,226]. In 2006, the first HSV v-miRNA was discovered. Currently, there are more than 27 known v-miRNAs that are encoded by HSV-1 and HSV-2 [226,238]. Recent studies have been focused on the detection of expressed miRNAs associated with latency-associated transcript (LAT) [226,246]. The expression of LAT is important in the maintenance of HSV-1 latency [228]. Infected cell polypeptide 4 (ICP4) is a regulator of viral transcription and is required for the productive infection of HSV-1 [236]. ICP0 is a viral immediate-early protein for which its expression determinates lytic viral replication. The expression of this protein is regulated by v-miRH2 in HSV-1-infected cells [237].

The first v-miRNA encoded by HCMV was identified in 2005, and so far, there are 26 known HCMV v-miRNAs [224,226]. One of the most studied HCMV v-miRNAs is miRUL112, and it can target both host and viral transcripts [246]. Major histocompatibility complex class I-related chain B (MICB) is a ligand recognized by NKG2D receptor expressed by CD8 T lymphocytes and NK cells, targeted by v-miRUL112 [224,228]. Toll-like receptors (TLR) play a key role in innate immune activation, as they recognize extracellular pathogens and present them to immune cells [238]. V-miRUL112-3p targets TLR2, leading to its silencing. The function of TLR2 is to bind to HCMV glycoproteins B and H, leading to proinflammatory cytokine production [238].

EBV genome harbors information for more than 44 miRNAs that have key roles in immune evasion, the inhibition of host adaptive immunity, or in the inhibition of host innate immunity [224,233]. The majority of these are transcribed from the BamH I-A rightward transcripts (BART) and BamH I-H right fragment 1 (BHRF1) regions [226,233]. V-miRBART2-5p suppresses hosts’ innate immune response [233].

KSHV-encoded miRK12-7 has the same function as EBV-encoded miRBART2-5p [228]. KSHV-infected endothelial cells overexpress v-miRK10a, which targets the TNF-like weak inducer of apoptosis receptor (TWEAKR). TWEAKR is a receptor for the proinflammatory cytokine TNF-like weak inducer of apoptosis (TWEAK) [228,238]. KSHV expresses a viral protein RTA that is essential for its viral phase, known as a master lytic switch [228]. RTA can be regulated directly or indirectly [224,228].

#### 3.1.2. Polyomaviruses

The most studied polyomaviruses include human polyomavirus BK (BKV), which causes polyomavirus-associated nephropathy and hemorrhagic cystitis, human polyomavirus JC (JCV), which causes progressive multifocal leukoencephalopathy, and the simian virus 40 (SV40) [224,247].

The two most-studied cellular miRNAs that are expressed during BKV infections are miR10 and miR30. The mechanisms of these two miRNAs are not yet fully discovered [239]. Bronchial epithelial cells infected by SV40 overexpress cellular miR27a [224]. BKV’s life cycle is mainly regulated by two BKV-encoded miRNAs, BKV-miRB1-5p and BKV-miRB1-3p, both of which are overexpressed during BKV infection [224,239]. JCV-encoded miRNAs that are overexpressed in the late phase of the infection are v-miRJ1-5p and v-miRJ1-3p [224,228].

The function of v-miRS1 encoded by SV40 has yet to be determined. Many studies suggest that its main function is the inhibition of expression of ING-4, a tumor suppressor that modulates p53, NF-κB, and HIF-1α activities [224,228].

#### 3.1.3. Papillomavirus

Human papillomaviruses (HPVs) are classified into two groups: low risk (LR-HPVs) and high risk (HR-HPVs) depending on their association with precursor lesions and their malignant potential, and among HR-HPVs, the most important types are HR-HPV HPV16 and HPV18 [218].

HPV16/HPV18-infected cells have a low expression of let-7 miRNA [240]. Another cellular miRNA that has an important role in HPV infection is miR125s [240]. Many HPV-encoded miRNAs are HPV16-encoded, and their functions have not been fully studied and understood [218]. Thus far, there are three main HPV miRNAs described, v-miR1, v-miR2, and v-miR3, that regulate the expression of genes involved in in cell migration and cell adhesion, such as *GATA6*, *ZEB2*, *THBS1*, and *STAT5B* [218].

#### 3.1.4. Adenoviruses

Adenoviruses not only mainly cause acute respiratory infections but are also associated with gastroenteritis, keratoconjunctivitis, myocarditis, meningoencephalitis, cystitis, and hepatitis [241]. During the first six hours of adenovirus infections, infected cells overexpress cellular miR22, which is a cell growth inhibitor, and a few tumor suppressor miRNAs such as miR181-b, miR320, or let-7. There is a second wave of cellular miRNAs expression twelve hours after the initiation of infection, which mainly consists of miRNAs with a role in immune response, such as miR29. [241].

Adenoviruses themselves encode two miRNAs: VA RNA1 and VA RNA2 [241]. VA RNAI produces the most prevalent mivaRNAs, mivaRNAI-137, and mivaRNAI-138, while VA RNAII produces a single mivaRNA and mivaRNAII-138 [226,241].

#### 3.1.5. *Hepadnaviridae*

Hepatitis B virus and hepatitis C virus (HBV and HCV, respectively) are the most studied hepatitis viruses and are known for liver infections. HBV modulates the host’s immune system by interacting with cellular miRNAs miR181 and miR155. In order to replicate and control host gene expression, HBV encodes two main v-miRNAs: v-miRNA-2 and v-miRNA-3 [242].

### 3.2. RNA Viruses

RNA viruses encode miRNA known as rv-miRNA. The functional role of these rv-miRNAs in the virus’ life cycle and in host cells is still not well understood. Some rv-miRNAs have extremally low levels of expression, making their detection methodologically challenging. Moreover, most RNA viruses replicate in the cytoplasm, and their viral miRNAs, therefore, do not interact with nuclear miRNA machinery [245].

#### 3.2.1. *Flaviviridae*

Hepatitis C virus, an RNA virus, along with hepatitis B virus is the most studied hepatitis virus [226,242,245].

The most studied cellular miRNAs for which its expression is deregulated during HCV infection are miR122 and miR155 [242,243]. It is believed that HCV does not encode rv-miRNAs [248].

#### 3.2.2. Retroviruses

The most studied retrovirus is human immunodeficiency virus 1 (HIV-1), and it infects T cells, especially CD4 T cells, and monocytes later in their differentiation [224].

HIV-1 can promote its replication by affecting cellular miRNAs [224,225]. Rv-miRN367 was the first-discovered HIV-1-encoded miRNA [224,226,245]. Another important HIV-1-encoded miRNA is rv-miRH1 [245].

#### 3.2.3. Influenza Virus

Influenza A virus (IAV) is the most known virus in this family. Contrary to other RNA viruses, IAV replicates inside the host’s nucleus and uses the host’s system to express its own rv-miRNAs [226,245].

H5N1, a subtype of IAV, causes different expressions of different miRNAs in infected cells [243,244]. H5N1’s first-observed encoded v-miRNA was miRHA-3p, and it regulates the expression of the *PCBP2* gene. This gene encodes the regulator of retinoic acid-inducible gene-1 and mitochondrial antiviral signaling (RIG-1/MAVS) [226,245].

#### 3.2.4. Coronaviruses

Coronaviruses are RNA viruses that cause severe acute respiratory syndrome, leading to high mortality [245]. Since 2020, due to the pandemic caused by COVID-19 infections, the interactions between cellular miRNAs and v-miRNAs have been meticulously studied [243]. To this date, 40 SARS-Cov-2-encoded miRNAs have been found, such as miR618, miR6501-5p, and miR144-3p. All miRNAs were upregulated in COVID-19-positive patients. Their immunological implication in the course of the infection is not yet fully understood, but a few studies suggest that these miRNAs function through NFκB, JAK/STAT or TGFβ signaling pathways [243].

## 4. Methods for miRNA Detection

As shown on the examples of cancer and viral diseases, miRNA have important roles in both cellular and physiological aspects in disease development and are, thus, emerging potential biomarkers. Being short, relatively unstable molecules, their detection is dependent on the type of tissue samples as well as on the sensitivity and precision of the method used for their evaluation. Methods adjusted for miRNA analysis that are mostly used so far are microarrays, quantitative real time polymerase chain reaction (qRT-PCR), in situ hybridization (ISH), Northern blotting (NB), and next-generation sequencing (NGS). Although all these methods have some (dis)advantages compared with others, they are all still used in different studies and can be used in miRNA detection assays. However, qRT-PCR and NGS are the two most commonly used methods in miRNA research and both have high potential in routine diagnostics applications [249].

### 4.1. Microarrays

Microarrays are commonly used for parallel analyses of a large number of known miRNAs. This method is based on the hybridization between target miRNAs and their complementary probes [250]. miRNA arrays differ in the oligo probe’s design, the probes immobilization chemistry, sample labelling, and microarray chip signal-detection methods [251]. Due to low abundance of miRNAs in total isolated RNA, it is necessary to enrich samples in miRNAs prior to the measurement of expression [252]. There are various technologies available for the labelling of target miRNAs, usually by a direct enzymatic labelling (e.g., T4 RNA ligase) [250], but target miRNAs can be detected even without being directly labelled [253]. This technology is relatively low-cost and enables a large number of samples to be processed in one experiment. Moreover, it can be used to relatively compare miRNA contents between two groups. Its disadvantage is that it cannot be used to detect new miRNAs.

### 4.2. Quantitative Real Time Polymerase Chain Reaction

qRT-PCR is a commonly used method for specific miRNA detection and absolute quantification of selected miRNAs [254]. miRNAs first need to be reverse-transcribed (RT) into complementary DNA (cDNA) using universal primers that require miRNAs to contain a polyA/poly U tail [255] or specific primers such as stem-loop RT primers [256], linear RT primers [257], DNA pincer probe [258], and two-tailed RT primers [259]. These extended requirements compared to standard RT primers are due to the length of miRNAs, which is the same as that of an average RT primer, and the fact that mature miRNAs contain the same sequences as their precursors (pre-miRNA and pri-mRNA) [259]. In order to distinguish between mature miRNAs and their precursors, hydrolysis probes are more often used in qPCR compared to intercalating dyes for the detection and quantification of miRNAs. The main advantages of this method are high sensitivity and specificity. On the other hand, only miRNAs with known sequences can be detected, and the reaction conditions are highly dependent on the contents of primers and probes.

### 4.3. In Situ Hybridization

Similarly to microarrays, ISH is also based on the hybridization between target miRNAs and their complementary probes, but while microarrays require extracted miRNAs, ISH is used to detect the presence of a specific miRNA directly in the cell/tissue of interest, enabling the localization of the targeted miRNA within the cell. By using specific probes marked with different fluorescent molecules, multiple miRNAs can be detected in one experiment [260].

### 4.4. Northern Blotting

NB is also based on the hybridization between target miRNAs and a complementary probe. Isolated RNA is first separated by size by denaturing gel electrophoresis. Two separate electrophoresis runs are performed in order to separate miRNAs from pre-miRNAs. miRNAs are subsequently transferred to a membrane, which is then probed with complementary oligonucleotides and left to hybridize over night. NB is highly specific and can determine the sequence and length of the target miRNA, which is the method’s greatest advantage. Both radioactive and non-radioactive probes can be used for the detection of miRNAs [261]. Susceptibilities to RNA degradation are the biggest disadvantage to this method, which is why it is less routinely used.

### 4.5. Next Generation Sequencing

NGS is currently the most often used method both for targeted miRNA detection and for the detection of novel miRNAs. It enables the processing of millions of sequences reads in a short period of time, while remaining highly sensitive and specific [262]. After RNA isolation, linkers are ligated to the 3′ and 5′-ends of RNAs, which are then reverse-transcribed, amplified using PCR, and sequenced [263]. Various software programs have been developed for the analysis of NGS data, usually specialized in specific analysis parts (isomiR detection and handling, exogenous sequences and different noncoding RNA detection, and de novo miRNA identification) [264]. These programs enable the retainment of reads relative to the length of miRNAs (15–40 nt); thus, the rest of the data pertaining mRNAs do not overwhelm the desired data [263]. Unlike the other methods used for miRNA detection, NGS is not limited to known miRNAs, which makes it the method of choice in most current studies. Although it is still relatively expensive and substantial computational support is needed for data analysis, it is the method that has the highest sensitivity and reproducibility [249,265,266].

## 5. miRNA Usage in Current Molecular Diagnostics

Based on the realization that miRNAs are often released in the bloodstream from different organs (brain, heart, endothelial cells, ovary, uterus, and mammary glands) and tumor tissues, miRNA diagnostic panels are designed to determine the cancer origin, disease progression, and chemoresistance prediction [267,268]. However, despite many published studies that show miRNA as a promising biomarker in personalized medicine, there are only a few panels used in clinical applications so far. One of the main obstacles is that when creating miRNA diagnostic panels, pre-analytical sample variables must be calculated in order to avoid a potential source of test inconsistency. Pre-analytical variables of both solid and liquid biopsy samples include sample quality or quantity of malignant cells, sample collection and preparation methods, type of fixative, and storage conditions [268]. Biopsy fluids have a major advantage over tissue biopsies because they are easier to obtain, especially in the case of urine and blood samples. Circulating miRNAs can be obtained from venous plasma or serum with no significant differences in their profiles when comparing these two types of blood samples [269,270]. Urine has some advantages compared to blood-based liquid biopsy, such as being non-invasive and easy to handle, but both samples are considered suitable for massive cancer screening based on miRNAs [269,270,271,272].

Due to challenges in developing accurate, simple, and commercially available diagnostic methods that involve standardized pre- and post-analytical procedures [268], there are only a few miRNAs’ panels offered to clinicians and that are covered by some insurance companies at the moment [273]. For clinical applications, the most important evaluation criteria for circulating miRNAs as diagnostic and prognostic biomarkers are high sensitivity and specificity, which are necessary to avoid false positive or negative diagnoses [268,272]. To our knowledge, only two companies have thus far developed a highly sophisticated and commercially available miRNA diagnostic test to detect genetic abnormalities within the thyroid nodule. ThyraMIR^®^ from Interspace Diagnostics, when used together with the ThyGeNEXT^®^ test [274] and RosettaGX Reveal^™^ test from Rosetta Genomics Ltd., is helpful in providing the most accurate information about the risk of an indeterminate thyroid nodule developing cancer [269,273,275,276]. The RosettaGX Reveal^™^ test produces the same high-level performance on ThinPrep-prepared slides as it does on a direct smear from a thyroid fine-needle aspirate biopsy [269,277]. The complementary use of molecular panels together with cytology enables differentiating between benign thyroid nodules that can be observed over time and malignant nodules that require surgery [275,276]. The second-generation miRview^®^ mets test from Rosetta Genomics can be used in identifying the primary origin of 42 tumors of uncertain or unknown origins, including sarcomas, lymphomas, and other non-epithelial malignancies [273]. The osteomiR^®^ test from TAmiRNA enables the parallel measurement of 19 microRNA biomarkers with respect to bone quality and osteoporosis in human serum samples [273,278]. ThrombomiRs^®^ tests from the same company enable an in vivo measurement of platelet functions, independently of the activation pathway as a part of the diagnostic procedure in cardiovascular diseases [273]. The Hummingbird Diagnostics developed miRNA diagnostics in the first two clinical applications, according to their website [279]. They developed a blood-based miRNA panel that detects the early stages of non-small cell lung cancer and predicts immunotherapy responses in stage IV of the disease.

At the moment, there is a trend in developing miRNA-based clinical panels, using blood samples as a noninvasive testing material [269], despite the fact that various biological fluids are suitable for miRNA isolation and detection [270,271,272]. The need for a quicker detection of different pathologic changes in humans also demands complex validation and standardization procedures before the application of miRNA detection panels in clinical practice in order to avoid potential technical biases [267,280]. Moreover, the application of miRNA detection in liquid biopsies for monitoring diseases is not very different from using cell-free DNA or circulating tumor cell (CTC) detection for the same purpose. All potential biomarkers that can be detected in body fluids have great potentials and some limitations, and they have been studied in recent years as panels that comprise the detection of mutation, methylation, and transcriptional regulation in the same test [281,282,283,284].

## 6. Future Perspective

As shown on the examples of cancer and infectious diseases, miRNAs have a very important role in disease development and progression. They govern intracellular processes and precisely regulate levels of expression of various cellular components; therefore, their deregulation contributes to pathogenesis. miRNAs are small molecules that regulate the expression of many genes with one very important specificity: miRNA families can have members with genes scattered across the entire genome, but they all have the same short nucleoid seed sequence (2–7 nt), and it is the main recognition sequence in targeting mRNAs, and one miRNA family can regulate hundreds of protein-coding genes [285]. Moreover, one miRNA regulates many different genes and processes, and most usually work in tandem with other miRNAs in forming panels that can be recognized as signatures of some diseases [286]. Because of these reasons, miRNAs are being explored as targets in diagnostics and/or personalized therapy. Firstly, they have been shown to have valuable diagnostics potential. They can be relatively easily detected and are used as a part of diagnostics criteria [280]; they have a potential for early cancer detection [287,288], and they can be obtained from both tissues and body fluids, which allows non-invasive early diagnostics [289,290,291,292,293]. Moreover, they can be used as targets in a specific approach in gene therapy. Their overexpression or downregulation associated with specific (sub)types of known diseases allows dual strategies in therapy: Their function can be substituted by using miRNA mimics or their functions can be diminished by using one of the approaches for their silencing, such as antagomirs or even molecular sponges [294,295,296]. When used as targets for sponges, the have an added value—they silence the entire miRNA family and, in that way, stop multiple changes in protein gene expression at once. Thus far, both strategies are explored, and although no miRNA-based therapy has yet been approved, nearly 100 are currently in clinical trials [297]. Finally, miRNAs-based gene therapy approach can be used as tuning tool for adjusting the level of drug metabolism [298]. However, although the role of miRNAs in disease development is undeniable because of their very complex mechanism of action, their usage as broadly used biomarkers is still under investigation.

Taken together, miRNAs are well-explored, thoroughly studied molecules that are, due to their availability and specificity, emerging markers with probable high potential not only in diagnostics but also in prognostic and in therapy procedures.

## Data Availability

Not applicable.

## Supplementary Materials

The following supporting information can be downloaded at: https://www.mdpi.com/article/10.3390/bioengineering9090459/s1, Table S1: Examples of the most investigated miRNAs in various tumors; Table S2: Host miRNAs in Herpesviridae infection; Table S3: Viral miRNAs in Herpesviridae infection; Table S4. Host miRNAs in polyomaviruses infection; Table S5, Viral miRNAs in polyomaviruses infection; Table S6: Host miRNAs in papillomavirus infection; Table S7: Host miRNAs in adenoviruses infection; Table S8: Host miRNAs in hepadnaviridae infection; Table S9: Viral miRNAs in hepadnaviridae infection; Table S10: Host miRNAs in RNA virus infection; Table S11: Viral miRNAs in RNA virus infection. Refs. [28,29,30,31,32,33,34,35,36,37,38,39,40,41,42,43,44,45,46,47,48,49,50,51,52,53,54,55,56,57,58,59,60,61,62,63,64,65,66,67,68,69,70,71,72,73,74,75,76,77,78,79,80,81,82,83,84,85,86,87,88,89,90,91,92,93,94,95,96,97,98,99,100,101,102,103,104,105,106,107,108,109,110,111,112,113,114,115,116,117,118,119,120,121,122,123,124,125,126,127,128,129,130,131,132,133,134,135,136,137,138,139,140,141,142,143,144,145,146,147,148,149,150,151,152,153,154,155,156,157,158,159,160,161,162,163,164,165,166,167,168,169,170,171,172,173,174,175,176,177,178,179,180,181,182,183,184,185,186,187,188,189,190,191,192,193,194,195,224,225,226,228,229,230,231,232,233,234,235,236,237,238,239,240,241,242,243,244,245] have been cited in supplementary materials.
