# Supplementary material for: miRNA in Molecular Diagnostics
Source: Bioengineering (Basel). 2022 Sep 9;9(9):459. doi: 10.3390/bioengineering9090459 (PMC9495386; doi:10.3390/bioengineering9090459)
Supplement: Supplementary file 1 [file bioengineering-09-00459-s001.zip › bioengineering-1844496-supplementary.pdf]

Table S1. Examples of the most investigated miRNAs in various tumors.

Type of tissue/tumor	miRNA	The role of miRNA	Genes and pathways	References
Chronic lymphocytic leukemia, Acute myeloid leukemia	miR15a, miR16	suppressor, apoptosis, proliferation	suppression of antiapoptotic BCL2 and MCL1, cyclin D1	[28–31]
B cell lymphomas, B cell leukemia	miR17-92 cluster: miR17, 18a, 19a, 20a, 19b-1, 92a-1	Oncomir, apoptosis, proliferation, survival, resistance to chemotherapy, BCR signaling	BIM, PTEN, p21 p57, CD22, PHLPP2, FCGR2B, ITIM, Bcl2 NFkappaB, PI3K pathways	[32–36]
B cell lymphoma, Chronic lymphocytic leukemia, pro/pre-B cells	miR34a	suppressor, apoptosis, proliferation	FOXP1, ZAP-70, BXL2, BCL6, B MYB, CDK6 and AXL	[37]
Mature B cells, mononuclear cells	miR150	survival, BCR signaling, immortality, migration	Myb, FOXP1, CXCR4, pathway PIK3AP1, AKT2	[26, 38, 39]
B cell lymphoma and leukemia	miR155	oncomir and suppressor, BCR activation, immunoglobulin somatic hypermutation, survival, proliferation, motility, modulation of TGFβ pathway	Pu.1 AID, SHiP-1, c/EBPβ, HGAL, RTKN2, SOCS, SHANK2	[40, 41]
Chronic lymphocytic leukemia	miR181b	suppressor, apoptosis, survival	Bcl2, MCL1, TCL1	[42, 43]
Lymphoma	miR21	oncomir, survival and chemotherapy resistance, proliferation, apoptosis, DNA repair	PTEN, PDCD4, hMSH2	[44, 45]
Lymphoma, Chronic lymphocytic leukemia	miR29a/b, c	suppressor, proliferation, apoptosis	CDK6, TCL1, MCL1	[26, 46]
Glioblastoma	miR21, miR338, miR485, miR491 and miR1290	apoptosis, proliferation	MAPK, PI3K/Akt, mTOR and Wnt signaling pathways	[47]
Glioblastoma	miR7	suppressor survival, proliferation, inhibition of apoptosis	Akt MAP kinase pathways	[48]
Glioblastoma	miR21	oncomir,	PI3K-Akt pathway,	[49–51]

		apoptosis, proliferation	PTEN, PDCD4, PDGF signaling	
Glioblastoma	miR451	migration, resistance to chemotherapy, survival	CAB39, AMPK pathway	[52, 53]
Glioblastoma	miR326	survival	Notch, PKM2	[54]
Glioblastoma	miR34a	proliferation, survival, migration, stemness, invasion	c-Met, Notch, CDK6, Akt and Wnt pathways	[55]
Glioblastoma	miR128	proliferation, self-renewal, stem cell maintenance, radioresistance	p53, RTK, Bmi-1, SUZ12 (PRC1 complex)	[56–58]
Glioblastoma	miR181	apoptosis, epithelial-mesenchymal transition	Bcl2, KPN4	[56, 59–61]
Glioblastoma	miR221, miR222	proliferation, DNA repair	p27, p57, MGMT	[56, 59, 62]
Lung carcinoma, small cell lung carcinoma	miR21	apoptosis, proliferation	PTEN in PI3K/Akt pathways, TGFβ pathway/SMAD7	[63, 64]
Small cell lung carcinoma	miR205	survival, proliferation migration	PHLPP2 in Akt signaling integrin α5	[65–67]
Small cell lung carcinoma	let-7	proliferation, cell cycle	Ras Myc pathways	[68, 69]
Lung carcinoma, non-small cell lung carcinoma	miR30	suppressor, apoptosis, survival, migration, epithelial-mesenchymal transition, resistance to chemotherapeutics	PI3K/Akt pathway p53, epithelial-mesenchymal transition	[70, 71]
Lung carcinoma	miR26	autophagy, apoptosis	TGFβ JNK signaling	[72]
Lung carcinoma	miR145	stemness, migration	epithelial-mesenchymal transition	[73]
Lung carcinoma	miR421	lipid metabolism	PTEN, PI3K/Akt/mTOR pathway signaling	[74]
Lung carcinoma	miR214	resistance to chemotherapy, angiogenesis, glycolysis, metastasis	PTEN/Akt/mTOR, PKM2, HK2, ING4, TWIST, HIF/VEGF pathways	[75, 76]
Non-small cell lung carcinoma	miR148a	apoptosis, epithelial-mesenchymal transition, invasion	MMP15, ROCK1	[77]
Non-small cell lung carcinoma	miR92a	invasiveness	RECK	[78, 79]

Breast carcinoma	miR125a	proliferation, drug sensitivity, autophagy	EGFR signaling, LIF receptor, Hippo signaling	[80, 81]
Breast carcinoma	miR145, miR21, miR155, miR205, miR125b	expression set		[82]
Breast carcinoma	miR205	proliferation, invasion	cdk2, cdk4, HMBG	[83, 84]
Breast carcinoma	miR153	angiogenesis	HIF/VEGFA pathway	[85]
Breast carcinoma	miR214	oncomir, suppressor, resistance to therapy invasion	resistance to chemotherapy, p53	[86, 87]
Breast carcinoma	miR21	apoptosis	PDCD4	[88]
Breast carcinoma	miR10b	metastasis	Twist-RHOC pathway	[89]
Breast carcinoma	let7	self-renewal, differentiation	Ras pathway, HMGA2	[90]
Breast carcinoma	miR210	hypoxia-dependent processes	HIF1	[91]
Breast carcinoma	miR205	proliferation, invasion, resistance to chemotherapy	HMGB3, VEGFA, FGF2	[84, 92]
Breast carcinoma	miR7	proliferation, apoptosis	Akt pathway	[93]
Bladder carcinoma	miR373	proliferation, migration and invasion	E cadherin, EGFR	[94, 95]
Bladder carcinoma	miR21, miR34a, miR222	cancer progression		[96–98]
Bladder carcinoma	miR223, 26b, 221, 103, 185, 23b, 203, 17, 23a, 205	upregulation		[99]
Bladder carcinoma	miR135	invasion	wnt/β-catenin pathway	[100]
Bladder carcinoma	miR31	chemotherapy resistance	integrin α5	[101]
Bladder carcinoma	miR200c, miR141, miR30b	set for invasive bladder tumor phenotype		[98]
Renal carcinoma	miR28, miR185, miR27, let7	upregulation		[99]
Renal carcinoma	miR21	proliferation	PTEN, mTOR signaling	[102]
Renal carcinoma	miR182	proliferation	Akt/FOXO3 signaling	[103]
Renal carcinoma	miR195	proliferation	VEGFR2, Pi3K/Akt pathway	[104]
Renal carcinoma	miR138	migration	SOX4, epithelial-mesenchymal transition, migration	[105]
Renal carcinoma	miR106b	proliferation	Wnt/β-catenin signaling	[106]
Renal carcinoma	miR203	proliferation, migration, apoptosis	GSK3β	[107]

Colorectal carcinoma	miR101	proliferation	COX2, Wnt/β-catenin	[108]
Colorectal carcinoma	miR141	proliferation	MAP kinase pathway	[109]
Colorectal carcinoma	miR200c	proliferation, migration	Wnt/β-catenin	[110]
Colorectal carcinoma	miR21	cell cycle progression, migration, invasion, resistance to chemotherapy	cdc25, hMSH2 TGFβ pathway	[45, 111, 112]
Colorectal carcinoma	miR362	cell cycle arrest	E2F1, USF2, PTPN1	[113]
Colorectal carcinoma	miR145	proliferation, migration, invasion	PAK4	[114]
Colorectal carcinoma	miR181	proliferation	NFκB signaling	[115]
Colorectal carcinoma	miR148b	proliferation, migration, invasion	Rock	[116]
Hepatocellular carcinoma	miR132	proliferation and apoptosis	PI3K/Akt PIK3R3	[117]
Hepatocellular carcinoma	miR194	invasion, migration, inflammation	NFκB pathway	[118]
Hepatocellular carcinoma	miR101	glucose metabolism	glycogen metabolism, PYGB	[119]
Hepatocellular carcinoma	miR485	migration, invasion	STAT3	[120]
Hepatocellular carcinoma	miR224	apoptosis	API5	[121]
Hepatocellular carcinoma	miR17-92, miR21	proliferation, anchorage, independent growth		[122]
Hepatocellular carcinoma	miR125	proliferation	Pokemon	[123, 124]
Hepatocellular carcinoma	miR18, 224	expression set		[125]
Gastric carcinoma	miR214	proliferation, migration, invasion	PTEN	[126]
Gastric carcinoma	miR101	migration	EZH2	[127]
Gastric carcinoma	miR370	proliferation	EGFR	[128]
Gastric carcinoma	miR138, miR204	proliferation, migration	EGFR	[129]
Gastric carcinoma	miR146a	proliferation, migration		[130]
Gastric carcinoma	miR223, 106b, 147, 34a,18a, 17, 98, 185, 638, 378	set of deregulated RNA		[131]
Gastric carcinoma	miR21	apoptosis	PDCD4	[132]
Gastric carcinoma	miR383	apoptosis	Bcl2	[133]
Gastric carcinoma	miR135b	proliferation, chemotherapy resistance	MAPK pathways	[134]
Pancreatic carcinoma	miR202	apoptosis, migration	TGFβ pathway, epithelial-mesenchymal transition	[135]
Pancreatic carcinoma	miR367	migration, invasion	TGFβ pathway, epithelial-mesenchymal transition	[136]
Cervical carcinoma	miR18a	proliferation	Hippo signaling	[137]

Cervical carcinoma	miR375	apoptosis, proliferation, chemoresistance	E cadherin	[138]
Cervical carcinoma	miR9	angiogenesis, migration		[139]
Cervical carcinoma	miR21	chemotherapy resistance	PTEN/Akt/HIF1, Akt/mTor pathway	[140]
Cervical carcinoma	miR138	proliferation, metastasis, epithelial-mesenchymal transition	EZH2	[141]
Prostate cancer	miR141	proliferation, migration, apoptosis	RUNX1	[142, 143]
Prostate cancer	miR182	invasion, proliferation	FOXF2, RECK, MTSS1	[144]
Prostate cancer	miR143, miR145	metastasis, apoptosis, proliferation, migration, invasion	epithelial-mesenchymal transition, PLD5	[145, 146]
Prostate cancer	miR205, miR31	resistance to chemotherapy	apoptosis	[147]
Prostate cancer	miR221, miR222	proliferation	p27Kip1	[148]
Prostate cancer	let7	chromatin modulation, growth, self-renewal	HMGA, androgen receptor signaling	[149]
Prostate cancer	miR21	apoptosis, invasion, angiogenesis	MARCKS, HIF and VEGF pathways, Akt MAPK pathways	[150, 151]
Testicular tumor: testicular germ cell tumor	miR371, miR372, miR373	cell cycle regulation, senescence	p53 pathway	[152–154]
Testicular germ cell tumor	miR223	apoptosis, cell growth	FBXW7	[155]
Testicular germ cell tumor	miR383	sensitivity to DNA damage	PNUTS	[156]
Testicular germ cell tumor	miR302	proliferation, invasion, apoptosis	MAPK PI3K/Akt signaling	[152, 157, 158]
Testicular tumor: seminomas and embryonal carcinoma	miR514a	apoptosis	NFκB, p53, Wnt pathways, apoptosis	[159]
Testicular tumor	miR199a	metabolism, gene expression	DNMT3, aerobic glycolysis	[160]
Skin tumor: melanoma	miR140	proliferation, invasion	WNT, NFκB signaling, SOX4	[161]
Melanoma	miR137		MITF, cell survival, chromatin remodulation	[162]
Melanoma	miR182	cell growth, migration, invasion	MITF, BCL2, cyclin D, Akt ERK pathways	[163]
Melanoma	miR148, miR137	proliferation	MITF	[164]
Melanoma	miR211	invasion, migration	AP1S2, SOX11, IGFBP5, SERINC3	[165]
Melanoma	miR7	proliferation, apoptosis	EGFR/IGF, PI3K/Akt signaling	[166]
Melanoma	miR211, miR221, miR10a	melanoma signature		[167]

Melanoma	miR155	immune surveillance	MITF	[168]
Melanoma	miR200c, 23b, 205, 146a, 155	melanoma signature		[169]
Skin tumor: cutaneous squamous cell carcinoma	miR125b	proliferation, invasion, migration	MMP13	[170]
Cutaneous squamous cell carcinoma	miR204	proliferation	MAPK, STAT3	[171]
Cutaneous squamous cell carcinoma	miR181a	proliferation	KRAS	[172]
Cutaneous squamous cell carcinoma	miR142	proliferation, stem-like properties	PTEN	[173]
Cutaneous squamous cell carcinoma	miR221	proliferation	PTEN	[174]
Cutaneous squamous cell carcinoma	miR203	proliferation, motility, angiogenesis	c-Myc	[175]
Cutaneous squamous cell carcinoma	miR148a	proliferation	MAP kinase pathway	[176]
Cutaneous squamous cell carcinoma	miR199a	proliferation, migration	CD44, metalloprotease activity	[177]
Cutaneous squamous cell carcinoma	miR124/214	proliferation	ERK pathways	[178]
Thyroid carcinoma: thyroid papillary carcinoma	mirR21, miR222, miR181b, miR146	proliferation, cell cycle	p27	[179–181]
Thyroid carcinoma	miR187, miR221, miR222, miR146b, miR155, miR224, miR197	thyroid tumor profile		[182]
Thyroid carcinoma	miR146	progression, proliferation, chemoresistance	KIT, RARB	[183, 184]
Thyroid carcinoma	miR30, miR200	proliferation, invasion, epithelial-mesenchymal transition	TGFβ pathway, epithelial-mesenchymal transition	[185, 186]
Thyroid carcinoma	miR145	migration, invasion	NFκB	[187]
Thyroid carcinoma: medullary thyroid carcinoma	miR10a, miR375	cell growth	YAP SLC16a2	[188]
Medullary thyroid carcinoma	miR183, miR375, miR9	profile		[189]
Medullary thyroid carcinoma	miR21	tumor progression	PDCD4	[190]
Osteosarcoma	miR17/92, miR20a	proliferation, migration	Β-catenin pathways, PTEN	[191]
Osteosarcoma	miR100, 125b, 22, 221/222, 27a, 29a	proliferation	IGF signaling	[192]
Osteosarcoma	miR192	proliferation, migration, apoptosis, invasion	TCF7	[193]
Osteosarcoma	miR23a	differentiation, proliferation	connexin 43, RUNX, CXCL12	[194, 195]

Table S2. Host miRNAs in Herpesviridae infection.

Virus	miRNA	Target	Regulation of target expression	Role	References
HSV-1	miR101	ATP5B	Downregulated	ATP5B depletion and blockage of HSV-1 DNA packaging and capsid maturation	[224]
		GRSF1	Downregulated	Binds to viral p40 mRNA, enhances its expression, and supports viral replication	[224]
	miR138	ICPO	Downregulated	Binds directly to its target and promotes viral latency	[224, 228]
	miR23	IRF1	Downregulated	Helps virus to avoid host immune system by damaging interferon pathways	[224]
	miR132	P300	Downregulated	Innate immune evasion	[224, 229]
HCMV	miR200 family	ULI122	Downregulated	Inhibition of viral reactivation	[224, 230]
	miR132	P300	Downregulated	Innate immune evasion	[224, 229]
EBV	EBNA1 protein upregulates Let-7a family	Dicer	Downregulated	Promotes EBV latency	[231]
	miR424	tumor suppressor gene SIAH1	Downregulated	Helps infected cells to avoid apoptosis	[224, 232]
	miRN17-92 cluster	PTEN and the pro-apoptotic protein Bim	Downregulated	Inhibits cellular differentiation and function, thus helping EBV escape host immune system	[233]
KSHV	miR608 and miR1293	vIL-6 and hIL-6	Downregulated	Evades host immune system by suppressing translation of vIL-6 and hIL-6	[224, 234]
	miR31	FAT4	Downregulated	Directly represses tumor suppressor and inhibitor of migration whose reduction enhances endothelial cell	[224, 235]

				mobility, leading to viral dissemination and angiogenesis	

Table S3. Viral miRNAs in Herpesviridae infection.

Virus	miRNA	Target	Regulation of target expression	Role	References
HSV-1	v-miRH6	ICP4	Downregulated	Leads to HSV-1 latency or suppression of viral production in infected cells	[236]
	v-miRNAH2	ICP0	Downregulated	Enhances latency of viral infection, increasing the amount of virus to be reactivated later during infection	[237]
HCMV	v-miRUL112	MICB	Downregulated	Makes the infected cells unrecognizable to NK cells and CD8 T cells	[224, 228]
	v-miRUL112-3p	TLR2	Downregulated	Significant for viral persistence as it prevents NFκB-mediated proinflammatory response	[238]
EBV	v-miBART2-5p	MICB	Downregulated	Makes the infected cells unrecognizable to NK cells and CD8 T cells	[233]
	v-miRBART1-35 and v-miRBHRF1-2-3p	IL-12B	Downregulated	Levels of IL-12 in infected B cells are decreased, leading to disruption of differentiation of CD4 T cells into T helper 1 cells and causing a reduction in cytotoxic T cells specific for resolving EBV infection	[233]
KSHV	v- miRK12-7	MICB	Downregulated	Makes the infected cells unrecognizable to NK cells and CD8 T cells	[228]

	v-miRK10a	TWEAKER	Downregulated	Reduces production of proinflammatory cytokines IL-8 and monocyte chemoattractant protein	[228, 238]
	v-miR (indirect effect), v-miRK12-7 and v-miRK12-9 (direct effect)	RTA	Downregulated	Leads to a prolonged latent phase of viral infection	[224, 228]

Table S4. Host miRNAs in polyomaviruses infection.

Virus	miRNA	Target	Regulation of target expression	Role	References
BKV	miR10<br>miR30	P53, bcl2-related protein A1, high temperature requirement protein A1 (HTRA1)	Unknown	Regulation of expression of genes involved in evading host immune response, preventing apoptosis and inducing inflammation	[239]
SV40	miR27a	IL-10	Upregulated	Enhances expression of proinflammatory cytokines in TLR2/4 activated macrophages that results in dysregulation of the cell cycle	[224]

Table S5. Viral miRNAs in polyomaviruses infection.

Virus	miRNA	Target	Regulation of target expression	Role	References
BKV	BKV-miRB1-5p<br>BKV-miRB1-3p	Large T antigen (LTAg)	Upregulated	Inhibition of LTAg expression, leading to prevention of viral replication and inability of cytotoxic T lymphocytes to recognize BKV-infected cells	[224, 239]
JCV	v-miRJ1-5p and v-miRJ1-3p	Large T antigen (LTAg)	Upregulated	Inhibition of LTAg expression, leading to	[224, 228]

				prevention of viral replication and inability of cytotoxic T lymphocytes to recognize BKV-infected cells	

Table S6. Host miRNAs in papillomavirus infection.

Virus	miRNA	Target	Regulation of target expression	Role	References
HPV16/HPV18	Let-7	Signal transducer and activator of transcription 3 (STAT3)	Downregulated	Overexpression of STAT3 results in increased inflammatory response	[240]
	miR125s	STAT3	Downregulated	Elevated matrix metalloproteinase 2 and 9 (MMP-2 and MMP9) leads to proinflammatory and anti-inflammatory impact during infection	[240]

Table S7. Host miRNAs in adenoviruses infection.

Virus	miRNA	Target	Regulation of target expression	Role	References
	VA RNA1	Protein double-stranded RNA-activated kinase (PKR)	Upregulated	Inactivates PKR, causing the inhibition of innate immune response	[241]
	mivaRNAI-137<br>mivaRNAI-138<br>mivaRNAII-138	Protein lymphocyte antigen 6 complex, Locus K (Ly6K), T-Cell-Restricted Intracellular Antigen-1 (TIA1)	Upregulated	Regulates cell growth and switching between early and late stage of infection	[226, 241]

Table S8. Host miRNAs in hepadnaviridae infection.

Virus	miRNA	Target	Regulation of target expression	Role	References
HBV	miR181	IL1-a	Upregulated	Has anti-inflammatory effect by inhibiting expression of IL1-a	[242]
	miR155	Suppressor of cytokine signaling 1 (SOCS1) CCAAT/enhancer binding protein (C/EBP)	Upregulated	Controls differentiation of T cells and is essential for normal B cell differentiation, leading to overactivation of immune system and impaired immune response	[242]

Table S9. Viral miRNAs in hepadnaviridae infection.

Virus	miRNA	Target	Regulation of target expression	Role	References
HBV	v-miR2	Ras-related nuclear protein (RAN)	Unknown	Regulation of RAN expression in other tissues correlates with cell proliferation, migration, and invasion	[226, 242]
	v-miR3	Unknown	Unknown	Correlates with HBV activity and enhances interferon (IFN) production	[242]

Table S10. Host miRNAs in RNA virus infection.

Virus	miRNA	Target	Regulation of target expression	Role	References
HCV (*Flaviridae*)	miR122	Exoribonuclease 2 (XRN2)	Downregulated	Needed for HCV replication, inhibits the interaction between XRN2 and HCV 5′UTR, protects the viral genome from degradation by XRN2	[242]
	miR155	Suppressor of cytokine signaling 1 (SOCS1)	Upregulated	Controls differentiation of T cells and is essential for normal B cell	[242, 243]

		CCAAT/enhancer binding protein (C/EBP)		differentiation, leading to overactivation of immune system and impaired immune response	
HIV-1 (*Retroviruses*)	miR132	Cellular transcriptional regulatory protein (MeCP2)	Upregulated	Downregulates expression of MeCP2, leading not only to promotion of viral replication, but also reactivation in monocytes	[224]
	miR221 and miR222	Receptor on CD4 T cells	Downregulated	Important for HIV-1 binding and upregulation of TNFα production, leading to restriction of entry of HIV-1	[224, 225]
H5N1 (*Influenza virus*)	miR29c	NFκB	Downregulated	Decreases the expression of NFκB pathway and inhibits host inflammatory response	[244]
	miR141	TGFβ2	Upregulated	Not fully understood. Presumed that it causes low host inflammatory response	[243, 244]

Table S11. Viral miRNAs in RNA virus infection.

Virus	miRNA	Target	Regulation of target expression	Role	References
HIV-1 (*Retroviruses*)	rv-miRN367	Suppressor factor gene Nef	Unknown	Leading to persistence of viral infection and survival of infected cells	[224, 226, 245]
	rv-miRH1	Apoptosis antagonizing transcription factor gene (AATF) and miRNA-140	Unknown	Inhibition and suppression of its targets leads to apoptosis inhibition and overexpression of viral accessory protein	[245]
H5N1 (*Influenza virus*)	miRHA-3p	Poly(rC)-binding protein 2 (PCBP2)	Upregulated	Downregulation of PCBP2 leads to cytokine storm	[226, 245]
